# Prediction of pre-eclampsia complicated by fetal growth restriction and its perinatal outcome based on an artificial neural network model

**DOI:** 10.3389/fphys.2022.992040

**Published:** 2022-11-17

**Authors:** Ke-Hua Huang, Feng-Yi Chen, Zhao-Zhen Liu, Jin-Ying Luo, Rong-Li Xu, Ling-Ling Jiang, Jian-Ying Yan

**Affiliations:** ^1^ Department of Obstetrics and Gynecology, Fujian Maternity and Child Health Hospital College of Clinical Medicine for Obstetrics and Gynecology and Pediatrics, Fujian Medical University, Fuzhou, China; ^2^ Graduate School of Fujian Medical University, Fuzhou, China

**Keywords:** pre-eclampsia, fetal growth restriction, peripheral blood parameters, perinatal outcomes, obstetrics and gynecology

## Abstract

**Objective:** Pre-eclampsia (PE) complicated by fetal growth restriction (FGR) increases both perinatal mortality and the incidence of preterm birth and neonatal asphyxia. Because ultrasound measurements are bone markers, soft tissues, such as fetal fat and muscle, are ignored, and the selection of section surface and the influence of fetal position can lead to estimation errors. The early detection of FGR is not easy, resulting in a relative delay in intervention. It is assumed that FGR complicated with PE can be predicted by laboratory and clinical indicators. The present study adopts an artificial neural network (ANN) to assess the effect and predictive value of changes in maternal peripheral blood parameters and clinical indicators on the perinatal outcomes in patients with PE complicated by FGR.

**Methods:** This study used a retrospective case-control approach. The correlation between maternal peripheral blood parameters and perinatal outcomes in pregnant patients with PE complicated by FGR was retrospectively analyzed, and an ANN was constructed to assess the value of the changes in maternal blood parameters in predicting the occurrence of PE complicated by FGR and adverse perinatal outcomes.

**Results:** A total of 15 factors—maternal age, pre-pregnancy body mass index, inflammatory markers (neutrophil-to-lymphocyte ratio and platelet-to-lymphocyte ratio), coagulation parameters (prothrombin time and thrombin time), lipid parameters (high-density lipoprotein, low-density lipoprotein, and triglyceride counts), platelet parameters (mean platelet volume and plateletcrit), uric acid, lactate dehydrogenase, and total bile acids—were correlated with PE complicated by FGR. A total of six ANNs were constructed with the adoption of these parameters. The accuracy, sensitivity, and specificity of predicting the occurrence of the following diseases and adverse outcomes were respectively as follows: 84.3%, 97.7%, and 78% for PE complicated by FGR; 76.3%, 97.3%, and 68% for provider-initiated preterm births,; 81.9%, 97.2%, and 51% for predicting the severity of FGR; 80.3%, 92.9%, and 79% for premature rupture of membranes; 80.1%, 92.3%, and 79% for postpartum hemorrhage; and 77.6%, 92.3%, and 76% for fetal distress.

**Conclusion:** An ANN model based on maternal peripheral blood parameters has a good predictive value for the occurrence of PE complicated by FGR and its adverse perinatal outcomes, such as the severity of FGR and preterm births in these patients.

## 1 Introduction

The incidence of pre-eclampsia (PE) is 2%–8% and results in 7%–8% of neonatal deaths or defects, (Ananth et al., 2013) accounting for 6% of maternal deaths during the perinatal period. Fetal growth restriction (FGR) is a serious complication of hypertensive disorder complicating pregnancy (HDCP), accounting for 42.3% of all perinatal deaths in China. (Vigil-De et al., 2003) Pre-eclampsia complicated by FGR may result in increased perinatal mortality. Barker’s (Barker et al., 1989) hypothesis suggests that FGR affects fetal, physical, and intellectual development in childhood and adolescence and significantly increases the incidence of adult metabolic syndrome. However, an early diagnosis of FGR is not easy, resulting in a relative delay in intervention and a lack of effective treatments. If a feasible prediction method can be found, it will be highly valuable in improving the outcomes of PE complicated by FGR.

Artificial neural network (ANN) models can predict complex relationships between variables that other models cannot compare, such as logistic regression models. Research in the biomedical field shows that the ANN is more accurate in classifying dependent variables than the logistic regression model. (Renganathan, 2019) Pre-eclampsia is affected by multiple factors, and common prediction method models cannot capture the complex cross-correlation of these factors. Artificial neural networks have been studied in PE diagnoses and delivery mode predictions. (Xu et al., 2019) Nair(Nair, 2018) constructed an ANN-based machine learning model using the identified biomarker microarray dataset and successfully classified PE and normotensive populations. The advantages of ANNs in dealing with these complex and heterogeneous features are confirmed again in the present study, which adopts the ANN to investigate the influence of maternal clinic characteristics and relevant blood parameters on pregnancy outcomes in PE complicated by FGR and to assess their value in predicting the occurrence of adverse perinatal outcomes.

## 2 Methods

### 2.1 Study population

For this retrospective descriptive study, all the subjects were singleton pregnancies with an accurate confirmation of their gestational age according to the last menstrual period and ultrasound results during early pregnancy. Those with pregnancy complications other than PE and FGR, those with unhealthy habits (such as substance abuse, smoking, and alcoholism), and those with incomplete medical records were excluded. A total of 270 pregnant patients with PE complicated by FGR (the concurrent group) who delivered in the Obstetrics Department of the Fujian Provincial Maternity and Children’s Hospital between January 2010 and December 2018 were selected as the study group. A total of 270 pregnant patients with PE not complicated by FGR (the PE group) and 270 pregnant patients with FGR but without PE (the FGR group) who were hospitalized during the same period were selected as the control groups, with a ratio of 1:1:1 between the three groups.

Substance abuse was defined as the repeated and heavy use of dependent substances unrelated to medical purposes, resulting in physical or mental dependence. (Ling, 1984) Alcoholism was defined as consuming more than 60 g of pure alcohol (defined in most countries as <6 standard glasses of white wine) in one sitting and having a blood-alcohol level of 0.08% or more. (WHO, 2014)

The Ethics Committee of the Fujian Provincial Maternity and Children’s Hospital approved the study and waived the need for informed consent, given its retrospective nature.

### 2.2 Data collection

Relevant clinical data were collected from the hospital’s electronic medical record system.

#### 2.2.1 General maternal information

This data included ages, places of residence, educational backgrounds, occupations, blood pressures, body masses, body mass indexes (BMIs) (calculated as weight [kg] ÷ height^2^ [m]), pre-pregnancy BMIs, gravidities, parities, gestational ages, medical histories, adverse pregnancies (and delivery histories), and family histories. Adverse pregnancy history included repeated abortions and a history of stillbirths. (Xie et al., 2018) Gestational age was determined according to the fetus size indicated by the last menstruation and early pregnancy color ultrasound. If the two were inconsistent, it was determined by the results of the early pregnancy color ultrasound.

#### 2.2.2 Relevant laboratory tests in late pregnancy (before delivery)

These tests included plasma glucose, lipid profiles, platelet parameters, hepatic and renal function, coagulation function, routine blood tests, and inflammatory parameters. Blood glucose, blood lipids, and liver and kidney function were measured after 8 h of fasting.

#### 2.2.3 Complications and pregnancy outcomes

The complications and outcomes recorded included hemolysis, increased liver transaminase-thrombocytopenia syndrome (HELLP), a premature rupture of membranes, placental abruption, postpartum hemorrhage, fetal distress, oligohydramnios, disseminated intravascular coagulation, admission to the intensive care unit, and gestational age at admission. Fecal contamination of amniotic fluid was defined as when the fetal anus relaxed and excreted feces, resulting in amniotic fluid contamination; this contamination was divided into three grades: grade I (light green), grade II (yellowish green with turbidities), and grade III (thick, tan color). (Xie et al., 2018) Placental adhesion was defined as when the placental villi adhered to the surface of the myometrium, mainly manifesting when the placenta failed to detach itself more than 30 min after delivery of the fetus. (Perlman et al., 2015) Intrahepatic cholestasis of pregnancy (ICP) was defined as a pregnancy-specific disease characterized by pruritus and elevated serum total bile acid levels. (Perlman et al., 2015) Umbilical cord torsion was defined as fetal activity causing the umbilical cord to twist along its longitudinal axis in a spiral shape longer than 16 weeks into pregnancy. (Xie et al., 2018) Placental abruption was defined as the placenta in its normal position after 20 weeks of gestation, partially or completely detaching from the uterine wall before delivery. (Xie et al., 2018) Placental abruption was graded by its severity: grade 0 = retrospective postpartum diagnosis after delivery; grade I = external blood without fetal distress; grade II = fetal distress or fetal death *in utero*; grade III = symptoms of maternal shock.

#### 2.2.4 Conditions of the placenta, fetus, and neonates

The conditions of the placenta, fetus, and neonates recorded included placental weight, placental size, placental pathological results, fetal distress, stillbirth, fetal malformation, neonatal birth mass, neonatal gender, neonatal length, Apgar score, transfers to the neonatal intensive care unit, neonatal respiratory distress syndrome, neonatal pneumonia, neonatal cerebral injury, and hemorrhage.

### 2.3 Diagnostic criteria

Pre-eclampsia diagnostic criteria: (Hypertensive Diseases in pregnancy and Chinese Society of Obstetrics and Gynecology, 2015) 1) systolic blood pressure ≥140 mmHg and/or diastolic blood pressure ≥90 mmHg for the first time after 20 weeks of gestation, accompanied by any one of the following: urine protein quantification of 300 mg/24 h, urine protein/creatinine ratio of 0.3, or random urine protein (+) (test method for unconditional urine protein quantification); 2) no urinary protein, but it was accompanied by any of the following organ or system disorders: thrombocytopenia (platelet count <100 × 10^9^/L); renal insufficiency (serum creatinine >97 umol/L or higher than two times the normal upper limit, excluding other kidney diseases; impaired liver function (transaminase two times higher than the normal upper limit); pulmonary edema; a new headache that common drug treatment did not alleviate, excluding other causes of visual impairment.

Fetal growth restriction diagnostic criteria: (American College of Obstericians and Gynecologists Committee, 2019) 1) prenatal ultrasound estimated that the fetal weight was lower than the 10th percentile of the normal weight for the same gestational age; (Zong et al., 2021) 2) the severity of lower-than-normal fetal weight (birth weight was lower than the third percentile of the normal weight for the same gestational age). Fetal weight was dynamically monitored by ultrasound every 2–3 weeks from 24 weeks of gestation until birth.

Postnatal asphyxia diagnostic criteria: (Perlman et al., 2015) 1) mild asphyxia (Apgar score—7 points in 1 min or seven points in 5 min); 2) severe asphyxia (Apgar score 3 points in 1 min or five points in 5 min).

### 2.4 Statistical methods

The SPSS 24.0 software was adopted for statistical analysis. The measurement data were expressed as the mean ± standard deviation or the median. An independent-sample *t*-test or non-parametric test was used to compare data between groups. Countable data were expressed as rates (%) and compared using a chi-squared test. *p* < 0.05 was considered statistically significant.

With the adoption of a three-layer (input, implicit, and output layers) ANN model (Liu, 2020), cases were randomly divided into training and testing sets with a ratio of 2:1. Factors with statistically significant differences between the two groups were used as input nodes of the ANN, perform data normalization preprocessing for the indicators with numerical quantities in them, and PE complicated by FGR and the adverse pregnancy outcomes were used as output nodes, the number of hidden layer nodes was according to the formula:m = √(n + l) +α, m Represents the number of hidden layer nodes, n Represents the number of nodes in the input layer, l Represents the number of nodes in the output layer, α Represents a constant between 1 and 10. We used Sigmoid as activation function. Six prediction models were then constructed, including the occurrence of PE complicated by FGR, postpartum hemorrhage, fetal distress, premature rupture of membranes, provider-initiated preterm births, and the severity of FGR were constructed. The trained neural network was tested for accuracy using the data in the testing set to evaluate the predictive value of ANNs.With the highest accuracy is considered as the best model, and calculated the F1 score, sensitivity (Sensitivity) and specificity (Specificity) of ANNs.

## 3 Results

**FIGURE 1:**
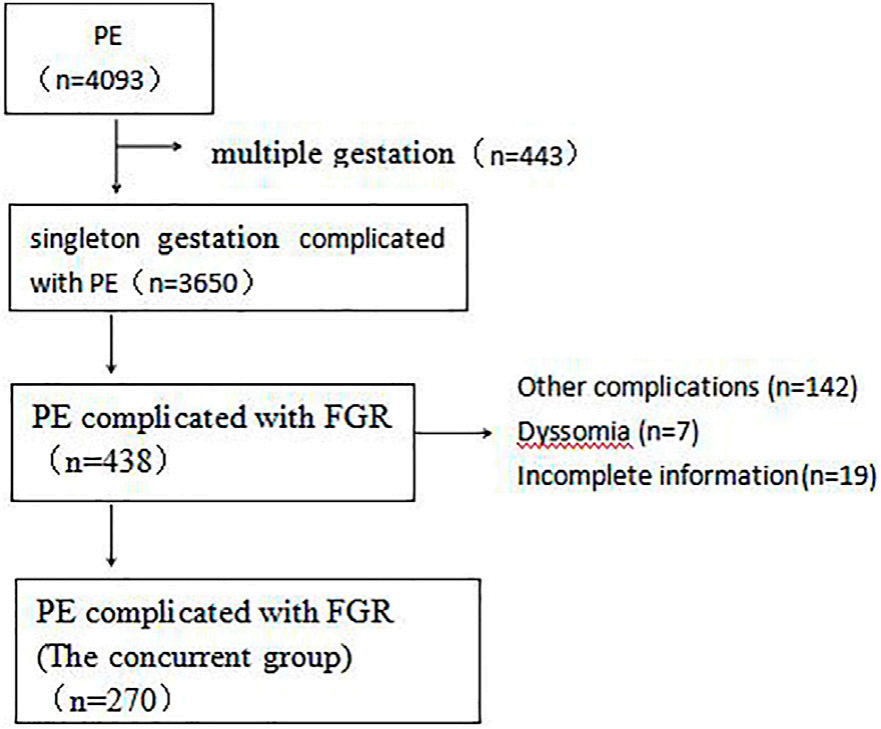
Selection flow chart of patients with preeclampsia complicated with FGR.

Table 1 presents the demographic and obstetric characteristics of the study population. The patients in the concurrent group had a higher pre-pregnancy BMI and were older than those in the PE and FGR groups. Women with more PE in prior pregnancy in the concurrent group complicated the FGR group. The differences between the three groups in adverse pregnancy and delivery gravity and parity were not statistically significant. The selection of research objects is shown in Figure 1.

**TABLE 1:** Comparison of the general characteristics among the pregnant women.

Groups	The concurrent group (*n* = 270)	The PE group (*n* = 270)	The FGR group (*n* = 270)	*p1*	*p2*
Pre-pregnancy BMI(Kg/m^2^)	21.62 ± 3.17	20.82 ± 2.69	19.39 ± 2.02	0.002	0.000
Age (Years)	29.51 ± 5.15	28.72 ± 3.83	27.8 ± 4.56	0.043	0.000
PE in prior pregnancy	38 (14.07%)	32 (11.85%)	5 (1.85%)	0.442	0.000
Adverse pregnancy and delivery	0.74 ± 1.00	0.62 ± 0.91	0.43 ± 0.84	0.158	0.100
gravidity	2.06 ± 1.18	2.00 ± 1.24	2.34 ± 1.42	0.120	0.202
parity	0.48 ± 0.59	0.35 ± 0.56	0.48 ± 0.62	0.150	0.212

Note: p1: Compared between the pre-eclampsia complicated with FGR, group and the pre-eclampsia without FGR, group; p2: Compared between the pre-eclampsia complicated with FGR, group and the FGR, group.

Analysis of maternal pregnancy outcomes showed that the incidences of fetal distress, placental adhesion, and postpartum hemorrhage were higher in the concurrent group than in the PE group. The incidences of HELLP, ICP, postpartum hemorrhage, premature rupture of membranes, placental abruption, placental adhesion, and fecal contamination of amniotic fluid were higher in the concurrent group than in the FGR group.

Analysis of neonatal–perinatal outcomes revealed that the incidences of prematurity, mild neonatal asphyxia, severe asphyxia, and admission to the newborn intensive care unit were higher in the concurrent group than in the PE and FGR groups. These differences were statistically significant (Table 2).

**TABLE 2:** Comparison of the incidence of complications in the pregnant women and the neonates [n (%)].

Groups	The concurrent group (*n* = 270)	The PE group (*n* = 270)	The FGR group (*n* = 270)	*p1*	*p2*
Abruptio placentae (grades 0,1)	14 (5.2%)	2 (0.7%)	7 (2.6%)	0.64	0
Abruptio placentae (grades 2,3)	8 (2.9%)	1 (0.3%)	0 (0%)	0.63	0
Meconium stained amniotic fluid	41 (15.1%)	50 (18.5%)	36 (13.3%)	0.05	0
Placental adhesions	5 (1.9%)	12 (4.4%)	2 (0.6%)	0	0.034
HELLP	17 (6.2%)	12 (4.4%)	3 (1%)	0.64	0
ICP	1 (0.5%)	1 (0.3%)	0 (0%)	0.63	0
Premature rupture of membranes	32 (11.8%)	38 (14.1%)	10 (3.6%)	0.442	0
Fetal distress	38 (14.1%)	8 (3.10%)	32 (11.8%)	0.049	0.442
Postpartum hemorrhage	25 (9.2%)	12 (4.4%)	2 (0.6%)	0	0
Torsion of cord	62 (22.9%)	65 (24.1)	111 (41.1)	0.063	0.155
Gestational age at delivery	35.81 ± 2.93	36.08 ± 9.10	39.05 ± 1.64	0.636	0.000
Premature newborn	150 (55.5%)	45 (16.7%)	21 (7.8%)	0	0
Mild neonatal asphyxia	5 (1.9%)	4 (1.48%)	5 (1.9%)	0.02	0
Severe neonatal asphyxia	4 (1.48%)	2 (0.6%)	0 (0%)	0	0.01
Mild small for gestational age	129 (47.8%)	0 (0%)	252 (93.2%)	0	0
Severe small for gestational age	141 (52.2%)	0 (0%)	18 (6.7%)	0	0
Admission to the NICU	168 (62.2%)	42 (15.5%)	62 (22.9%)	0	0
Stillbirth	2 (0.6%)	1 (0.4%)	0 (0%)	0.11	0.062
Neonatal respiratory distress syndrome	9 (3.33%)	8 (2.96%)	6 (2.22%)	0.56	0.44
Neonatal pneumonia	6 (2.22%)	4 (1.48%)	5 (1.85%)	0.34	0.21
Neonatal cerebral injury	8 (2.96%)	4 (1.48%)	3 (1.11%)	0.31	0.12
Neonatal cerebral hemorrhage	7 (2.59%)	4 (1.48%)	3 (1.11%)	0.29	0.11

Note: p1: Compared between the pre-eclampsia complicated with FGR, group and the pre-eclampsia without FGR, group; p2: Compared between the pre-eclampsia complicated with FGR, group and the FGR, group.

The following hematological parameters with statistically significant differences were screened by intergroup difference analysis (*p* < 0.05): inflammatory parameters (neutrophil-to-lymphocyte ratio [NLR] and platelet-to-lymphocyte ratio [PLR]), coagulation parameters (prothrombin time [PT] and thrombin time [TT]), lipid parameters (high-density lipoprotein [HDL], low-density lipoprotein [LDL], and triglyceride [TG]), platelet parameters (platelet count [PLT], mean platelet volume [MPV], and plateletcrit [PCT]), uric acid (UA), lactate dehydrogenase (LDH), and total bile acids (TBA). All these parameters could be used as input nodes for the ANN as the basis for constructing the ANN models, as illustrated in Table 3.

**TABLE 3:** Comparison of the hematological parameters among pregnant women.

Groups	The concurrent group (*n* = 270)	The PE group (*n* = 270)	The FGR group (*n* = 270)	*p1*	*p2*
PLT (×10^9^/L)	205.89 ± 63.38	210.66 ± 68.18	210.80 ± 52.04	0	0.009
MPV (%)	11.79 ± 7.22	10.10 ± 1.75	10.74 ± 1.06	0.001	0
PCT (%)	0.20 (0.16,0.25)	0.23 (0.19,0.27)	0.23 (0.19,0.25)	0	0
PT (s)	12.89 ± 0.38	11.66 ± 0.18	11.00 ± 1.04	0	0
APTT (s)	28.79 ± 3.22	27.80 ± 4.75	27.74 ± 4.06	0.1	0.22
TT (s)	16.21 ± 3.25	15.98 ± 3.20	14.97 ± 2.97	0	0
FDP (μg/ml)	12.36 ± 3.43	8.40 ± 6.59	9.94 ± 10.26	0.12	0.1
FIB (g/L)	4.26 ± 1.26	4.53 ± 4.79	4.57 ± 0.81	0.1	0.21
D-Dimers	4.33 ± 1.27	4.04 ± 3.95	3.22 ± 2.48	0.12	0
Fasting blood glucose during late pregnancy	5.00 ± 1.40	4.92 ± 1.24	4.84 ± 1.17	0.456	0.151
OGTT0 (mmol/L)	4.31 ± 0.31	4.39 ± 0.35	4.30 ± 0.32	0.721	0.673
OGTT1 (mmol/L)	7.46 ± 0.62	7.65 ± 0.59	7.53 ± 0.63	0.101	0.258
OGTT2 (mmol/L)	5.71 ± 0.56	5.66 ± 0.51	5.75 ± 0.53	0.236	0.471
ALT (U/L)	19.00 (13.40,28.00)	13.00 (9.20,20.30)	14.10 (10.20,21.30)	0.061	0.063
GGT (U/L)	15.90 (12.00,31.00)	11.00 (9.30,18.30)	12.00 (10.00,18.00)	0.102	0.092
Uric Acid	407.00 (345.00,472.80)	367.00 (294.00,426.50)	299.05 (244.80,347.95)	0	0
AST (U/L)	26.66 ± 28.44	23.14 ± 14.00	20.03 ± 8.46	0.068	0.001
LDH (U/L)	352.58 ± 223.84	295.47 ± 151.95	330.94 ± 192.25	0.002	0.241
Total bile acid	3.82 ± 2.77	4.51 ± 3.72	3.98 ± 2.71	0.016	0.008
ALB (g/L)	31.11 ± 4.31	33.20 ± 18.17	33.77 ± 4.45	0.103	0
PLR	149.88 ± 138.44	131.55 ± 46.84	108.78 ± 58.03	0.004	0.002
NLR	4.79 ± 2.58	4.32 ± 2.21	4.15 ± 1.82	0.002	0.003
TC (mmol/L)	3.75 ± 1.78	3.53 ± 1.20	2.93 ± 1.17	0.12	0
TG (mmol/L)	6.54 ± 1.78	6.21 ± 1.23	5.81 ± 1.09	0.02	0
Apo-A1 (g/L)	1.67 ± 0.26	1.62 ± 0.25	1.73 ± 0.19	0.052	0.003
Apo-B (g/L)	1.34 ± 0.38	1.27 ± 0.37	1.30 ± 0.30	0.051	0.268
HDL (mmol/L)	1.85 ± 0.45	2.06 ± 0.63	2.09 ± 0.42	0	0.592
LDL (mmol/L)	3.82 ± 1.07	3.24 ± 1.08	2.97 ± 0.88	0	0

Note: p1 Compared between the pre-eclampsia complicated with FGR, group and the pre-eclampsia without FGR, group; p2 Compared between the pre-eclampsia complicated with FGR, group and the FGR, group. Due to the skewed distribution of ALT, GGT, and uric acid, the medians (P25, P75) were used for statistical descriptions and the rank-sum test was adopted for statistical testing.

PLT:platelet count,PDW:platelet distribution width, APTT:activated partial thromboplastin time,FDP:fibrinogen degradation products,FIB:fibrinofen, OGTT:Oral glucose tolerance test,Alt:alanine aminotransferase,Ast:aspartate transaminase,GGT:r-glutamyltransferase,LDH:lactic dehydrogenase,ALB:albumin,TC:total cholesterol,TG:triglyceride,Apo-A1:apolipoprotein A1; Apo-B:apolipoprotein B.

Cases were randomly divided into training and testing sets with a ratio of 2:1. A total of 15 parameters, including the serological parameters that were statistically different between groups and the maternal ages and pre-pregnancy BMIs, were used as input nodes to construct the ANN model. The Batch_Size was selected as 10. and the learning rate was set to 0.3. After experiment, the optimal number of hidden layer nodes was selected. The number of hidden layer nodes in each model was 10 for preeclampsia complicated with fetal growth restriction model, eight for postpartum hemorrhage model, eight for fetal distress model, eight for premature rupture of membranes model, eight for preterm delivery model, and 10 for FGR severity model. The trained ANN was tested for accuracy using the data in the testing set. The accuracy of the ANN for predicting the occurrence of the following diseases and adverse outcomes was as follows: 84.3% for PE complicated by FGR, 81.9% for predicting the severity of FGR, 80.3% for premature rupture of membranes, 80.1% for postpartum hemorrhage, 77.6% for fetal distress, and 76.3% for provider-initiated preterm delivery. The details are shown in Table 4.

**TABLE 4:** BP artificial neural network models and the top five influencing factors of weights.

Pregnancy outcome	Influencing factors	Weight	Accuracy (%)	Sensitivity (%)	Specificity (%)	F1 score
Pre-eclampsia complicated with FGR	NLR	3.06	84.3	97.7	78	0.81
	HDL	2.98				
	Pre-pregnancy BMI	2.95				
	MPV	2.65				
	LDH	1.98				
Postpartum hemorrhage	PLR	2.78	80.1	92.3	79	0.31
	Age	2.54				
	TT	2.24				
	PT	1.63				
	TG	1.53				
Fetal distress	LDH	3.17	77.6	92.3	76	0.44
	Age	3.01				
	PLR	3.00				
	Pre-pregnancy BMI	2.55				
	Total bile acid	2.54				
Premature rupture of membranes	Pre-pregnancy BMI	4.72	80.3	92.9	79	0.50
	Age	3.44				
	TG	3.23				
	MPV	2.95				
	PCT	2.82				
Provider-initiated preterm births	Age	2.93	76.3	97.3	68	0.69
	HDL	2.87				
	LDL	1.49				
	Pre-pregnancy BMI	1.48				
	PT	1.38				
Severity of SGA	LDL	3.63	81.9	97.2	51	0.88
	NLR	3.54				
	HDL	3.48				
	Pre-pregnancy BMI	3.44				
	MPV	2.34				

## 4 Discussion

Pre-eclampsia is a disease peculiar to pregnancy. The perinatal mortality rate is increased, and it is closely related to the short-term and long-term complications of the mother and child. Because an early diagnosis of FGR is not easy, it is very important to study and predict the risk factors of PE complicated by FGR. This study retrospectively analyzed the clinical data and hematological parameters, such as inflammatory, coagulation, and blood lipid indexes, and the platelet parameters of pregnant women in PE complicated by FGR. There were 15 risk factors of PE complicated by FGR screened out, and a prediction model for predicting disease occurrence and adverse perinatal outcomes was established by using an ANN. This study analyzed the clinical characteristics and perinatal outcomes of PE complicated by FGR. The results showed that the age and pre-pregnancy body mass index of women with PE complicated by FGR were larger than those of women with PE or FGR alone. The incidences of fetal distress, placental adhesion, postpartum hemorrhage, preterm delivery, and mild and severe neonatal asphyxia were significantly higher in patients with PE complicated by FGR than in those with only PE or FGR. These results confirm that women with a high body mass index before pregnancy and older women were more likely to have PE complicated by FGR. The perinatal outcomes in patients with PE complicated by FGR were worse than in those with only PE or only FGR. Savitri et al. (Savitri et al., 2016) also found that the pre-pregnancy BMI, rather than the weight gain during pregnancy, determined the level of blood pressure during pregnancy and was related to the higher probability of PE and its complications. However, the close relationship between older age and PE is well known. Another study found that the older age of pregnant women is related to uterine and placental dysfunction; (Lean et al., 2017) this should be the key to the adverse consequences of FGR and its effects on the fetus caused by the aging process. The most common cause of FGR is placental insufficiency due to impaired placental blood flow and vascular abnormalities, (Alfaidy et al., 2014; Alfaidy et al., 2020), (Alfaidy et al., 2014; Alfaidy et al., 2020) which is similar to the pathogenesis of PE. Therefore, patients with PE are prone to FGR, which aggravates the original pathological state and causes the perinatal outcome of the mother and child to be worse than with PE or FGR alone.

Maternal serological parameters changed markedly with the onset and progression of PE. This study found that 13 parameters, including blood lipid indexes (HDL, LDL, TG), coagulation indexes (PT, TT), platelet parameters (PLT, MPV, PCT), inflammatory indexes (NLR, PLR), uric acid, LDH, and total bile acid were related to PE complicated by FGR. Furthermore, the levels of TG and LDL were higher, and the HDL level was lower in patients with PE complicated by FGR than in those with only PE, and the differences were statistically significant. The mechanism for this might be because increased serum TG concentrations and increased LDL production during pregnancy cause increased calcium levels in vascular smooth muscle cells, raise the degree of oxidative stress, and injure the vascular endothelial cells, thus triggering PE. (Goulopoulou and Davidge, 2015) Furthermore, dyslipidemia disrupts endothelial cell function and stimulates endothelial cells to synthesize more thromboxane, which in turn causes blood vessels to spasm. This would lead to shallow chorionic villus implantation, which can reduce blood flow to the placenta and uterus and impede nutrient supply and fetal development, which can further induce the occurrence of FGR in patients with PE.

The results of the oral glucose tolerance test during mid-pregnancy and the fasting plasma glucose during late pregnancy were compared among the three groups in the present study. The results showed that the differences were not statistically significant. The reason for these outcomes might be correlated with the fact that patients with gestational diabetes mellitus or a pre-pregnancy history of diabetes mellitus were excluded from the present study. Whether glucose metabolism influences the occurrence of PE complicated by FGR, and the mechanisms involved, requires further investigation.

Platelet activation may occur during the early stage of PE. (Kohli et al., 2016; Armalyz et al., 2018) The results of the present study showed that PLT in patients with PE complicated by FGR was lower than in those with only PE and that MPV and platelet distribution width (PDW) were higher, and the differences were statistically significant (*p* < 0.05). This may be correlated with platelet aggregation and depletion due to injury to the vascular endothelium in patients with PE complicated by FGR. This aggregation and depletion would cause the average PDW to increase due to the reduced platelet count, resulting in increased blood viscosity and the potential for microthrombosis. Microcirculatory disorders might also lead to ischemia and hypoxia, which could further affect the growth and development of all organs and the fetus. (Kurtoglu et al., 2015) The present study also found that PT and TT in patients with PE complicated by FGR were lower than in those with only PE or only FGR, while D-dimer levels were higher in patients with PE complicated by FGR than in those with only FGR, and the differences were statistically significant (*p* < 0.05). Shortened activated partial thromboplastin time and PT are closely correlated with a hypercoagulable state and represent a higher likelihood of thrombosis, leading to multiple thromboses, reduced fibrinolytic activity, and impaired ability to repair endothelial cells.

The occurrence of PE is mainly correlated with elevated levels of the inflammatory response. (Zhao et al., 2017) The platelet-to-lymphocyte ratio is closely correlated with immune response and can be considered a class of immune response factors. (Jeon et al., 2017) The neutrophil-to-lymphocyte ratio is strongly correlated with cardiovascular diseases (such as coronary heart disease), inflammatory diseases, and obstetric and gynecological malignancies. (Myatt and Cui, 2004) The results of the present study provide further evidence that inflammatory immune mechanisms might play a role in disease progression in patients with PE.

Most current prediction studies are based on logistic regression models to predict only FGR or PE. (Yu et al., 2017; Saleh et al., 2021; Feng et al., 2022) Most of these studies were single-index predictive analyses, such as the glucose and lipid metabolism index or single coagulation index, with unsatisfactory sensitivity and specificity. For example, Ren Danyu et al. (Ren and Wang, 2019) analyzed the coagulation function index and its application value in judging the severity of PE, and the area under the receiver operating characteristic curve was 0.67 (*p* > 0.05). Few studies have been conducted on creating a predictive model for FGR in patients with PE. Artificial neural networks are distributed parallel information-processing algorithmic mathematical models that imitate the behavioral characteristics of animal neural networks. This type of network relies on the complexity of the system to process information by adjusting the interconnectivity between a large number of internal nodes. Wang et al. (Wang et al., 2016) used logistic regression and ANN models to evaluate the high-risk factors of PE before and during pregnancy and compared the efficiency of the two in diagnosing PE. They found that the fitting effect of the ANN in grading PE in the third trimester of pregnancy is more suitable for multivariate analysis of complex diseases due to logistic regression, and the efficiency of ANN in disease diagnosis and prediction has also been confirmed in many studies. (Ibrahim et al., 2022; Miao et al., 2022)

At present, relevant data from ultrasounds are used in predicting PE and have proven to be valuable. (Lai et al., 2022) Ultrasound measurement is considered a bone marker, neglecting soft tissues, such as fetal fat and muscle, and the cross-section selection and fetal position’s influence will lead to estimation errors. The pathophysiological changes of PE are systemic multi-system and multi-organ damage, which will cause harm to the mother and child. Ultrasound generally monitors the changes in the fetus and uterus, which may occur later than the hematological parameters and other indicators reflecting the state of systemic diseases. Therefore, selecting clinical characteristics and hematological parameters for prediction is more meaningful than ultrasounds. The present study combined ANNs, interdisciplinary cooperation, and an artificial intelligence system to build an effective and simple predictive model based on hematological parameters and clinical characteristics of pregnant women. The internal verification showed that the ANN accuracy rate of predicting PE complicated by FGR is 84.3%, the accuracy rate of predicting FGR severity is 81.9%, and the accuracy rate of predicting preterm birth is 76.3%. Moreover, these models all have a good F1 score (this value balances the recall and accuracy of the model and is a good evaluation index of the ANN model). In addition, the ANN model for predicting postpartum hemorrhages, premature rupture of membranes, and fetal distress has a good accuracy rate. However, due to the influence of the basic incidence rate of disease, the data groups are in an unbalanced state, and the accuracy and F1 scores of the model are low, though this cannot deny the value of the model.

## 5 Conclusion

In pregnant patients with PE, monitoring the changes in relevant hematological and metabolic parameters during pregnancy offers a useful guide to predicting the development of FGR and improving perinatal outcomes. The present predictive model, combining coagulation parameters, lipid metabolism, and inflammatory hematological parameters, has a good predictive value in predicting the occurrence of PE complicated by FGR and pregnancy complications, such as postpartum hemorrhage and fetal distress.

## Data Availability

The original contributions presented in the study are included in the article/supplementary material further inquiries can be directed to the corresponding author.

## References

[B1] Alfaidy N. Brouillet S. Rajaraman G. Kalionis B. Hoffmann P. Barjat T. (2020). The emerging role of the prokineticins and homeobox genes in the vascularization of the placenta: Physiological and pathological aspects. Front. Physiol. 11, 591850. 10.3389/fphys.2020.591850 33281622 PMC7689260

[B2] Alfaidy N. Hoffmann P. Boufettal H. Samouh N. Aboussaouira T. Benharouga M. (2014). The multiple roles of EG-VEGF/PROK1 in normal and pathological placental angiogenesis. Biomed. Res. Int. 2014, 451906. 10.1155/2014/451906 24955357 PMC4052057

[B3] American College of Obstericians and Gynecologists Committee (2019). Fetal growth restriction ACOG practice bulletin number 204. Obstet. Gynecol. 133 (2), e97–e109. 10.1097/AOG.0000000000003070 30681542

[B4] Ananth C. V1 Keyes K. M. Wapner R. J. (2013). Pre-eclampsia rates in theUnited States, 1980-2010:age-period-cohort analysis. BMJ 347 (15), f6564. 10.1136/bmj.f6564 24201165 PMC3898425

[B5] Armalyz J. Jabbour A. Abassi Z. A. (2018). Preeclampsia: Novel mechanisms and potential therapeutic approaches. Front. Physiol. 9, 973. 10.3389/fphys.2018.00973 30090069 PMC6068263

[B6] Barker D. J. Osmond C. Golding J. Kuh D. Wadsworth M. E. (1989). Growth *in utero*, blood pressure in childhood and adult life, and mortality from cardiovascular disease. BMJ 298, 564–567. 10.1136/bmj.298.6673.564 2495113 PMC1835925

[B7] Feng Y. Zheng H. Fang D. Mei S. Zhong W. Zhang G. (2022). Prediction of late-onset fetal growth restriction using a combined first- and second-trimester screening model. J. Gynecol. Obstet. Hum. Reprod. 51 (2), 102273. 10.1016/j.jogoh.2021.102273 34813940

[B8] Goulopoulou S. Davidge S. T. (2015). Molecular mechanisms of maternal vascular dysfunction in preeclampsia. Trends Mol. Med. 21 (2), 88–97. 10.1016/j.molmed.2014.11.009 25541377

[B9] Hypertensive Diseases in pregnancy and Chinese Society of Obstetrics and Gynecology (2015). Guidelines for diagnosis and treatment of hypertensive diseases during pregnancy. Chin. J. Obstetrics Gynecol. 50 (10), 721–728.

[B10] Ibrahim Z. Tulay P. Abdullahi J. (2022). Multi-region machine learning-based novel ensemble approaches for predicting COVID-19 pandemic in Africa. Environ. Sci. Pollut. R., 1–23. 10.1007/s11356-022-22373-6 PMC9365685 35948797

[B11] Jeon Y. Lee W. I. Kang S. Y. Kim M. H. (2017). Modified complete blood count indices as predicting markers of preeclampsia from gestational hypertension:neutrophil to lymphocyte ratio,platelet to lymphocyte ratio,and. Clin. Lab. 63, 1897. 10.7754/Clin.Lab.2017.170705 29226641

[B12] Kohli S. Ran Jan S. Hoffmann J. Kashif M. Daniel E. A. Al-Dabet M. M. (2016). Maternalextracellular vesicles and platelets promote preeclampsia viainflammasome activation in trophoblasts. Blood 128 (17), 2153–2164. 10.1182/blood-2016-03-705434 27589872

[B13] Kurtoglu E. Kokcu A. Celik H. Sari S. Tosun M. (2015). Plateletindices may be useful in discrimination of benign and malign endometrial lesions, and early and advanced stageendometrial cancer. Asian pac. J. Cancer Prev. 16 (13), 5397–5400. 10.7314/apjcp.2015.16.13.5397 26225684

[B14] Lai J. Syngelaki A. Nicolaides K. H. von Dadelszen P. Magee L. A. (2022). Using ultrasound and angiogenic markers from a 19- to 23-week assessment to inform the subsequent diagnosis of preeclampsia. Am. J. Obstet. Gynecol. 227 (2), 294.e1–294.e11. 10.1016/j.ajog.2022.03.007 35276067

[B15] Lean S. C. Heazell A. E. P. Dilworth M. R. Mills T. A. Jones R. L. (2017). Placental dysfunction underlies increased risk of fetal growth restriction and stillbirth in advanced maternal age women. Sci. Rep. 7 (1), 9677. 10.1038/s41598-017-09814-w 28852057 PMC5574918

[B16] Ling G. M. (1984). The global drug abuse problem: Analysis and perspectives. Influ. Sci. Soc. (01), 8–16.

[B17] Liu R. (2020). An overview of the basic principles of artificial neural networks. Comput. Prod. Circulation (06), 3581–3635.

[B18] Miao J. Wei Z. Zhou S. Li J. Shi D. Yang D. (2022). Predicting the concentrations of enteric viruses in urban rivers running through the city center via an artificial neural network. J. Hazard. Mat. 438, 129506. 10.1016/j.jhazmat.2022.129506 35999718

[B19] Myatt L. Cui X. (2004). Oxidative stress in the placenta. Histochem. Cell Biol. 122 (4), 369–382. 10.1007/s00418-004-0677-x 15248072

[B20] Nair T. M. (2018). Statistical and artificial neural network-based analysis to understand complexity and heterogeneity in preeclampsia. Comput. Biol. Chem. 75, 222–230. 10.1016/j.compbiolchem.2018.05.011 29859381

[B21] Perlman J. M. Wyllie J. Kattwinkel J. Wyckoff M. H. Aziz K. Guinsburg R. (2015). Neonatal resuscitation: 2015 international consensus on cardiopulmonary resuscitation and emergency cardiovascular care science with treatment recommendations (reprint). Pediatrics. 10.1542/peds.2015-3373D 26471381

[B22] Ren D. Y. Wang Y. H. (2019). Study on the auxiliary diagnostic value of coagulation function and platelet parameters in preeclampsia and its severity. Chin. general Pract. 22 (22), 2698–2704.

[B23] Renganathan V. (2019). Overview of artificial neural network models in the biomedical domain. Bratisl. Lek. Listy 120 (7), 536–540. 10.4149/BLL_2019_087 31602991

[B24] Saleh L. Alblas M. M. Nieboer D. Neuman R. I. Vergouwe Y. Brusse I. A. (2021). Prediction of pre-eclampsia-related complications in women with suspected or confirmed pre-eclampsia: Development and internal validation of clinical prediction model. Ultrasound Obstet. Gynecol. 58 (5), 698–704. 10.1002/uog.23142 33030757 PMC8596877

[B25] Savitri A. I. Zuithoff P. Browne J. L. Amelia D. Baharuddin M. Grobbee D. E. (2016). Does pre-pregnancy BMI determine blood pressure during pregnancy? A prospective cohort study. BMJ Open 6 (8), e011626. 10.1136/bmjopen-2016-011626 PMC4985806 27515754

[B26] Vigil-De G. P. Montufar-Rueda C. Ruiz J. (2003). Expectant management ofsevere preeclampsia and preeclampsia superimposed on chronichypertension between 24 and 34 weeks' gestation. Eur. J. Obstet. Gynecol. Reprod. Biol. 107 (1), 24–27. 10.1016/s0301-2115(02)00269-5 12593889

[B27] Wang H. M. Shen T. Zeng Z. J. (2016). The comparative study on the efficiency of BP neural network and Logistic regression grading diagnostic model. J. Trop. Med. 16 (03).

[B28] WHO (2014). Global status report on alcohol and Health 2014[R]. Geneva: WHO.

[B29] Xie X. Kong B. H. Duan T. (2018). Obstetrics and Gynecology. 9th Ed. Beijing: People's Medical Publishing House, 138.

[B30] Xu Y. Feng Q. Chang H. L. (2019). Glucolipid metabolism in preeclampsia complicated with gestational diabetes mellitus and its effect on neonates. Chin. Matern. Child. Health Care 34 (24), 5623–5624.

[B31] Yu N. Cui H. Chen X. Chang Y. (2017). First trimester maternal serum analytes and second trimester uterine artery Doppler in the prediction of preeclampsia and fetal growth restriction. Taiwan. J. Obstet. Gynecol. 56 (3), 358–361. 10.1016/j.tjog.2017.01.009 28600048

[B32] Zhao K. Xiang B. X. Q. Rong W. H. (2017). Analysis and clinical significance of platelet and coagulation indexes in patients with severe preeclampsia in Xizang Plateau. Jilin Med. J. 38, 2217–2219.

[B33] Zong X. N. Li H. Zhang Y. Q. Wu H. H. (2021). [Reference values and growth curves of weight/length, body mass index, and ponderal index of Chinese newborns of different gestational ages]. Chin. J. Pediatr. 59 (03), 181–188. 10.3760/cma.j.cn112140-20201130-01063 33657691

